# Charnley femoral cemented stem with a permeable and resorbable cement restrictor and low-viscosity cement: Clinical and radiographical evaluation of 100 cases at a mean follow-up of 6.55 years

**DOI:** 10.1051/sicotj/2019034

**Published:** 2019-11-01

**Authors:** Jean-Louis Prudhon, Jacques H. Caton, Thierry Aslanian

**Affiliations:** 1 Centre Osteo Articulaire 5 rue Raoul Blanchard 38000 Grenoble France; 2 Institut d’orthopédie 103 rue Coste 69300 Caluire France; 3 Consultant 25 chemin jan Baptiste Gillard 69300 Caluire et cuire France

**Keywords:** Resorbable and permeable cement restrictor, Cementation, Cardiovascular side effects

## Abstract

*Introduction*: In 1979, in his first book dealing with low-friction arthroplasty (LFA), Charnley highlighted the use of a cement restrictor. Breusch and Malchau described in 2005 the “second-generation cementing technique.”

The main objective of this study was to report on the clinical survival of 100 cases of Charnley femoral component implanted in 2007 and 2008 using a permeable and resorbable cement restrictor and a low-viscosity antibiotic-loaded cement.

The secondary objectives were to analyze the complications and side effects and the accuracy of the device positioning.

*Material and methods*: This was a monocentric retrospective review of a prospectively compiled database.

Diaphyseal restrictor was biodegradable and permeable to gas, blood, and fluids to avoid intramedullary over pression during cementation. The cement was a low-viscosity antibiotic-loaded cement.

Among 3555 patients, we selected the first continuous 100 cases of patients where we implanted the device.

Survival probability was computed according to Kaplan–Meier method.

*Results*: Mean follow-up was 6.55 ± 2.6 (range 1–11).

Considering femoral component revision as the endpoint, survival rate was 100%.

No patients died intraoperatively, none in the first month and the first year after surgery.

No early periprosthetic fractures have been reported.

*Discussion:* As described initially by Charnley, the use of a cement restrictor was highly recommended through the different generations of cementing techniques.

Hypotensive episodes and cardiac arrest have been reported during cement insertion.

In our series, we did not deplore any adverse effect related to the cementation.

*Conclusion*: Our study demonstrates a 100% survival rate of a cemented femoral component without adverse effects when using routinely a resorbable and permeable cement restrictor and a low-viscosity cement. Bone cement is still a fantastic ally for the surgeon and the patients.

## Introduction

Cemented femoral component in total hip arthroplasty (THA) has been our standard since 1982.

Cementing technique originally described by J Charnley [1] has improved over year.

**Figure 1:**
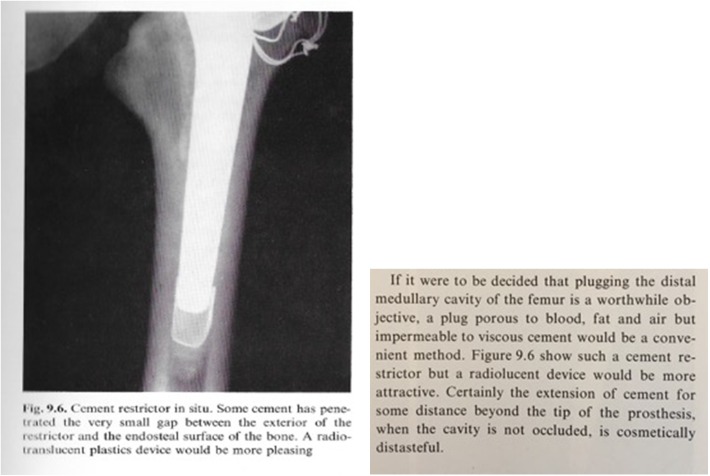
Pictures from LFA J Charnley (1979, p. 115).

In 1979, in his first book dealing with low-friction arthroplasty (LFA), Charnley highlighted the use of a cement restrictor, and obviously, he described a resorbable and permeable “cement restrictor” (Figure 1).

Breusch and Malchau described in 2005 [2] the “second-generation cementing technique.” It included the use of low-viscosity cement mixed under vacuum, introduced with a cement gun, a diaphyseal cement restrictor, and a washing technique to clear out blood during cementation.

We had initially in 2002 [3] reported on the efficiency of a resorbable and permeable restrictor on cementing femoral component and prevention of cardiovascular disorders.

The main objective of this study was to report at a mean follow-up (fu) of 6.55 years, on the clinical survival of 100 cases of Charnley femoral component (Institution^™^ Groupe Lepine, Genay, France) implanted in 2007 and 2008 in our institution using a permeable and resorbable restrictor Air Plug^™^ cement restrictor (APCR) and a low-viscosity antibiotic-loaded cement (Aminofix 3^™^).

The secondary objectives were to analyze

1. what were the complications (early, midterm, late) and causes for revision,
2. the accuracy of the APCR positioning and the quality of the cement mantle at the latest fu.

## Material and methods

This was a monocentric retrospective review of a prospectively compiled database (FileMaker Pro). This clinical study was conducted in compliance with good clinical practice, in accordance with the Declaration of Helsinki, and in all applicable regulatory requirements including national laws.

### Diaphyseal restrictor

Air plug was made of gelatin, glycerol, injectable water, *p*-hydroxybenzoate of methyl. The APCR was flexible enough to adapt to irregularities in shape of the femoral canal to ensure adequate occlusion.

**Figure 2:**
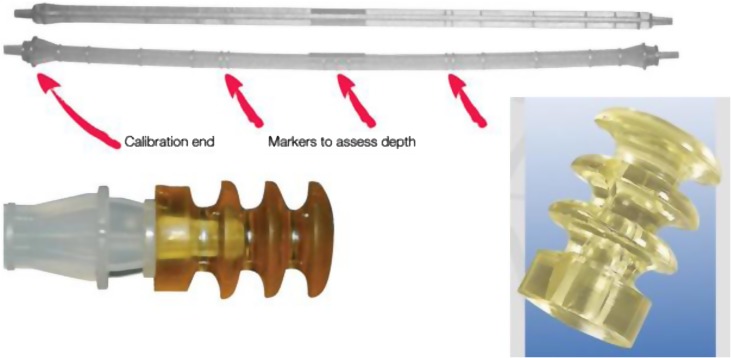
Single use ancillary device for APCR implantation.

- Its biodegradable nature eliminated the need to extract it at a possible revision of the femoral stem.
- A vent system was achieved through a set of slits arranged in staggered rows (scattered slits).
- Cylindrical, it is composed of three collars of the same diameter and a cylindrical base of a smaller diameter.
- The specific shape of the obturator (slits and collars) contributed to blocking the flow of cement between the collars and gives optimum stability during pressurization of the cement.
- The base of the plug was drilled to facilitate holding and positioning in the diaphyseal canal with the help of an obturator holder.
- The APCR fit to uneven diaphyseal canal due to its shape and flexible structure.
- The slits made the obturator permeable to gas, blood, and irrigation fluids.
- It made it possible to avoid an intramedullary overpressure during the introduction of the femoral stem in the diaphysis canal (Figure 2).

### Low-viscosity cement

Aminofix 3^™^ was a bone low-viscosity acrylic cement for fixation of joint prosthesis. It is made of a liquid component mainly composed of a monomer of methyl methacrylate and a powder component of polymethyl methacrylate. It was opacified by barium sulfate (near than 10% in the powder component) and loaded with gentamicin sulfate (1 g/dose).

The preparation on the operative theater was obtained by mixing liquid and powder components in compliance with the requirements of the manufacturer.

### Femoral component

Institution^™^ femoral stem was made of stainless steel M30NW. The angle between the shaft and the neck was 130°. A modular head could be used (small, medium, long, metallic 22.2 or 28, ceramic 28 thanks to a Morse taper). The neck was circle, and highly polished to decrease impingement and stresses on the acetabular cup.

### Acetabular cup

The acetabular cup used in this series was a regular polyethylene cementless press-fit cup (RM Mathys^™^).

In two cases, a dual-mobility cup (ADES Dedienne) was used.

### Patients

Among 3555 patients collected in our database (1988–2015), we have routinely used the APCR in 1872 patients since 1998.

From April 2007 to April 2008, we have selected the first continuous 100 cases of patients where we implanted the Institution^™^ femoral component, cemented with Aminofix 3^™^ cement and APCR. No patients were excluded.

The characteristics of the cohort are reported in Table 1.

**Table 1:** Characteristics of the cohort.

Number of patients	100
Gender: female/male	51/49 (51%)
Mean age at surgery (years)	62.98 ± 11.15
Preoperative ASA score	
1–2	81 (81%)
3–4	4 (4%)
Not reported	15 (15%)
Preoperative Devane score	
Devane 2–3 (active and very active)	95 (95%)
Devane 4 (sedentary)	5 (5%)
Devane 5 (dependant)	0
Not reported	3 (3%)
Weight/BMI status	
Normal (20–25)	59 (59%)
Mild obesity (26–30)	23 (23%)
Severe obesity (31–35)	15 (15%)
Charnley classification	
Class A	36 (36%)
Class B	56 (56%)
Class C	4 (4%)
Aetiology	
Osteoarthritis	87 (87%)
Hip dysplasia	2 (2%)
Rhumatoid arthritis	4 (4%)
Fracture	0
Post traumatic arthritis	0
Osteonecrosis	7 (7%)
Bearing	
Ø 28 Ceramic on polyethylene (CoP)	78
Ø 22.2 metal on polyethylene (MoP) Stainless steel head	22

### Operative technique

All the THAs were performed through a posterolateral approach with a capsular repair.

The APCR was inserted in the medullary canal with a plastic single-use introducer after checking the size of the diameter canal (Figure 2).

In our technique, we attempted to put the APCR close to the tip of the femoral component. In such a situation, the tip of the femoral implant, after resorption of the APCR, was free of cement, and some micromotion of the implant inside the cement mantle was possible.

The low-viscosity cement was introduced in an anterograde manner, with a cement gun in the medullary canal.

To avoid the risk of cardiovascular collapse, the cement was prepared as recommended by the manufacturer: mixing polymer and powder during 45 s in a plastic bowl (atmospheric conditions), 45 s of complete phase rest, cement pouring in the gun, injection after a minimum of 8 min according to the viscosity.

The anesthesiologist was always present with the patient in the operative room at that stage of the procedure to manage blood pressure, heart rate, blood oxygen saturation.

Patients were recommended to walk with immediate full weight bearing with two crutches for a couple of weeks. They regularly discharged at 7 days.

Post-op X-ray was undertaken in the recovery room.

An outpatient visit with a clinical examination and X-ray was routinely performed at 4 months, 1 year, and every 2 years.

All the data were collected in computerized software (FileMaker Pro).

### Outcomes evaluation

- Intraoperative mortality and adverse event, immediate post-op mortality, morbidity (infection, deep venous thrombosis, periprosthetic fracture, hematoma, and all adverse events) were recorded.
- Radiographical evaluation included frontal plane X-ray of the pelvis and frontal and sagittal plane X-ray of the hip. The implant position, the cement mantle quality, and the position of the plug were analyzed. Radiolucent sign, migration or subsidence of the implants, fracture of the plug, wear of the cup, complications, and revision were collected.

Three different positions of the plug were described:

Position 1: No cement at the tip of the stem.
Position 2: 1 cm or less thickness of the cement between the plug and the tip of the stem.
Position 3: More than 1 cm of cement over the tip of the stem (Figure 3).
  **Figure 3:**
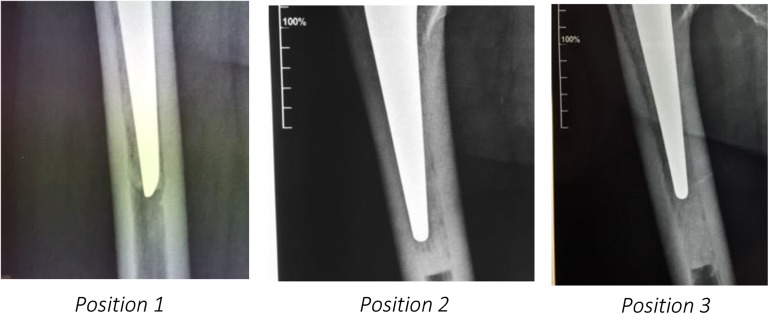
X-ray evaluation of APCR position related to the tip of the stem.

A close analysis of zone 3–5 was done to assess bone remodeling, sclerosis, or lysis at 1 year and at the latest follow-up.

### Statistical analysis

Qualitative variable was presented as percentage, quantitative variables as mean or median, standard deviation and range. Survival probability, considering revision surgery of the stem for any reason as endpoint, was computed according to Kaplan–Meier method with Stata software.

## Results

Nine patients were lost for follow-up.

Three (four hips) patients died for causes unrelated to the hip surgery.

### Survival

Mean follow-up was 6.55 ± 2.6 (range 1–11).

Considering femoral component revision as the endpoint, survival rate was 100%.

**Figure 4:**
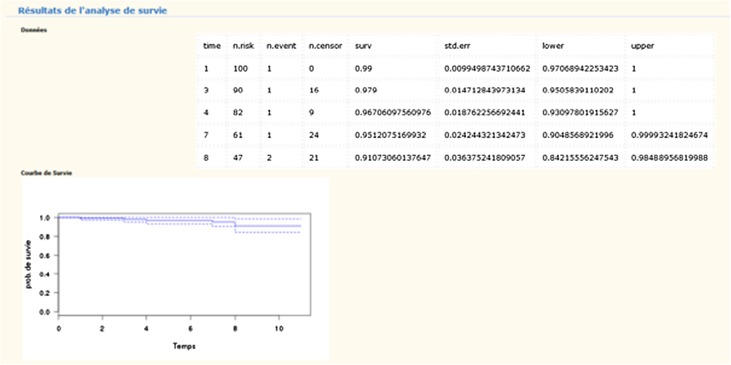
Kaplan–Meier survival curve.

Considering revision for any cause as the endpoint, survival rate is 91.07% at 11 years with an interval confidence of 95% [84.21–98.49] (Figure 4).

### Mortality and morbidity

No patients died intraoperatively, none in the first month and the first year after surgery.

No early periprosthetic fractures have been reported.

In two cases, a deep wound infection occurred at 3 weeks and 6 weeks after surgery. In both cases, a lavage was performed and antibiotics were used for 3 months; at the latest follow-up, implants are still in place, and the patients are doing well and have not been revised (Table 2).

**Table 2:** Mortality, morbidity, complications.

Mortality, morbidity	
Mortality	0
Immediate post-op complications	
Early infection	2
Early dislocation (<1 year)	4
Late dislocation	4
Peri-prosthetic fracture	1
Haematoma	1
Deep vein thrombosis	1
Revision	
Minor revision (deep wound infection)	2
Major revision	6
Acetabular revision for dislocation	6
Femoral revision (loosening)	0

One patient (73-year-old BMI = 40.78, osteoarthritic hip) developed on Day 6 a DVT and was successfully treated medically.

### Complications

Six major revisions have been performed: six isolated acetabular revisions for recurrent instability.

No femoral revision for femoral loosening was reported. One patient has presented a periprosthetic femoral fracture (Vancouver B1) at 7 years. He was treated with open reduction and internal fixation with a cerclage. At the latest follow-up, the femoral implant was still in place with no sign of loosening (Table 2).

### Accuracy of the APCR positioning

According to the operative technique previously described, our target was to implant the APCR in position 1. The goal was obtained in 75 cases. At the latest follow-up, the APCR has totally disappeared (Figure 3).

**Figure 5:**
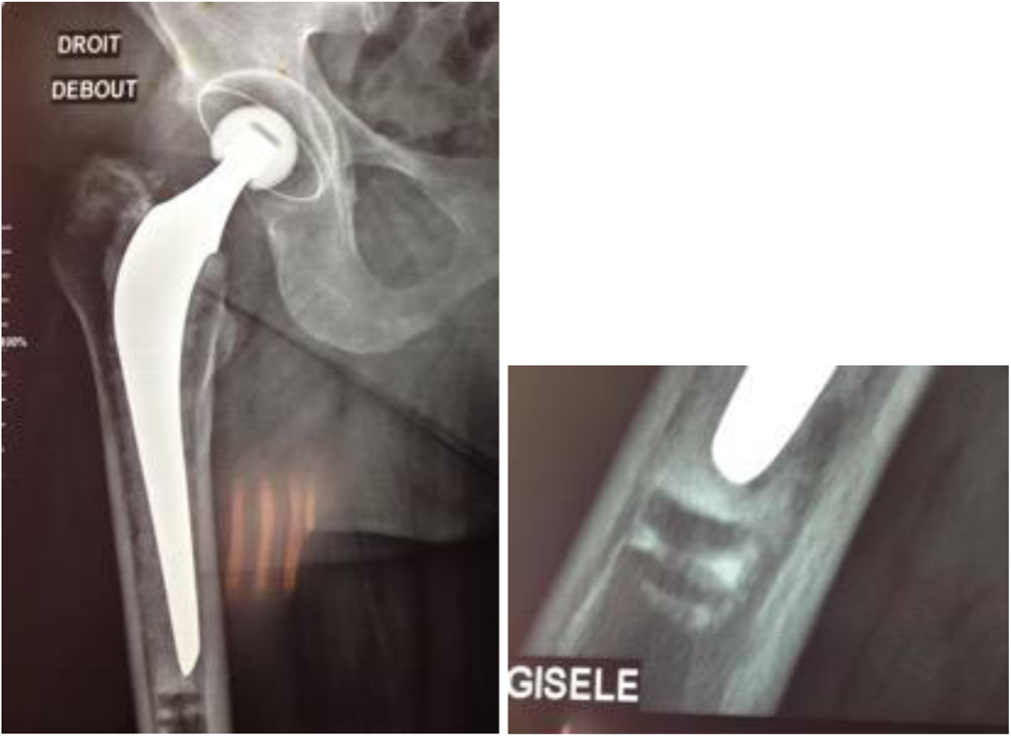
APCR position 2. Traces of cement in APCR are still visible.

In 25 cases, the APCR was in position 2, and at the latest follow-up, it was easy to recognize the traces of the plug penetrated by the cement (Figure 5). In two of these cases, the cement tip fractured in the first 3 months without any clinical damage.

In four cases, the APCR had significantly migrated during surgery.

Modification of the bone in zone 3 has been observed in 1 case.

## Discussion

### Limitations of the study

It is a monocentric retrospective study of a single surgeon. The sample size is limited; results presented in this study are midterm and long-term results.

#### Evolution of cementing techniques

Advancements in cementing can be classified to have occurred from “first-generation” to “third-generation techniques,” with changes occurring in bone bed preparation, cement preparation, and cement delivery [4].

As described initially by Charnley [1], the use of a cement restrictor was highly recommended whatever the nature of the plug was. Its use has always been recommended through the different generations of cementing techniques [5–9].

##### First-generation cementing technique

It involved the hand mixing of cement. There was only a minimal preparation of the femoral canal, and cancellous bone was left in situ. The canal was irrigated and suctioned prior to the digital application of cement. The prosthesis was then inserted into the femoral canal. Pressurization of the cement was introduced to improve cement diffusion into cancellous bone (osseointegration of the cement), and the importance of a good cement mantle around the prosthesis was more clearly understood.

##### Second-generation cementing techniques

All cancellous bone was removed and distal cement restrictor was also systematically used. A pulsatile irrigation allowed a fair cleaning of the femoral canal. The cement was inserted with a cement gun.

Ilizaliturri et al. [10] reported on plug migration and cement mantle assessment in THA. They concluded that clinical outcomes are not influenced by the position of the plug.

In our series, there were no differences in the outcomes between the three positions we have identified.

#### Fatal risk with the use of low-viscosity cement

Hypotensive episodes and cardiac arrest have been reported during cement insertion [11].

Pressurization and thorough cleaning of the bone with expulsion of bone marrow have been associated with the occurrence of pulmonary embolisms, and this risk has been found to be increased in patients with osteoporotic bone and femoral neck fracture.

The premature insertion of bone cement may lead to a fall in blood pressure, which has been linked to the availability of methyl methacrylate at the surface of the product [12]. This fall in blood pressure can lead to cardiac arrhythmias or to an ischaemic myocardium. However, the possible risk of death associated with the use of cemented implant is confined to early postoperative and perioperative.

We had initially in 2002 reported on the efficiency of a resorbable and permeable restrictor on cementing femoral component and prevention of cardiovascular disorders [3]. Pitto et al. [13, 14] had previously reported on the prevention of cardiovascular side effects by using a vacuum cementation of the femoral component.

Assi et al. [15] had reported a low mortality rate with cemented Charnley femoral component and dual-mobility cup in a frail population of femoral neck fracture.

In our series, we did not deplore any intraoperative or early adverse effect related to the cementation. One patient has presented a DVT detected by ultrasonography (US) on Day 6 (the first US control on Day 2 was negative).

This type of permeable cement restrictor seems a reliable prevention of severe adverse effects during femoral cementation.

#### Long-term outcomes of cemented THA implants

Since 1982, we have been using LFA. More than 4000 THAs have been implanted. In our experience, long-term outcomes [16, 17] are consistent with that of reported by numerous authors [16–23] and data from national registries [24].

Cemented LFA is the basis of the modern THA.

Abdel et al. [25] had reported a 40-year observational study of 2000 patients treated with the Charnley cemented THA to analyze what is the lifetime risk of revision for patients undergoing THA. The authors concluded that the Charnley cemented THA provided excellent long-term results at 40 years. Their results and analysis provide a benchmark for comparison by the subsequent changes in the design of THAs.

The weak point of LFA was the acetabulum. Dislocation was one of the main early complication. Polyethylene wear was the second main concern in the long-term follow-up. However, cementation has been a dramatic improvement in the field of joint replacement.

Cement is not responsible for the failures of poor design implants or surgical errors. It does not kill the patients as reported sometimes. It must be used in respect of the rules of cementation recommendations.

Bone cement is still a fantastic ally for the surgeon and the patients.

## Conclusion

Our study demonstrates a 100% survival rate of a cemented femoral component without intraoperative or early adverse effects when using routinely a resorbable and permeable cement restrictor and a low-viscosity cement.

## Conflicts of interest

Jean Louis Prudhon recieves Royalties from Groupe lepine. Jacques Caton has no disclosure. Thierry Aslanian has no disclosure.
